# Efficacy of adjuvant chemotherapy according to hormone receptor status in young patients with breast cancer: a pooled analysis

**DOI:** 10.1186/bcr1778

**Published:** 2007-10-11

**Authors:** Jos A van der Hage, J Sven D Mieog, Marc J van de Vijver, Cornelis JH van de Velde, cooperating investigators of the European Organization for Research and Treatment of Cancer

**Affiliations:** 1 Leiden University Medical Center, Department of Surgery, Albinusdreef 2, 2333 ZA Leiden, The Netherlands; 2 European Organization for Research and Treatment of Cancer Data Center, Avenue Mounierlaan, 83/11 Brussel 1200 Bruxelles, Belgium; 3 The Netherlands Cancer Institute, Department of Pathology, Plesmanlaan 121, 1066 CX Amsterdam, The Netherlands

## Abstract

**Introduction:**

Breast cancer at a young age is associated with an unfavorable prognosis. Very young patients with breast cancer therefore are advised to undergo adjuvant chemotherapy irrespective of tumor stage or grade. However, chemotherapy alone may not be adequate in young patients with hormone receptor-positive breast cancer. Therefore, we studied the effect of adjuvant chemotherapy in young patients with breast cancer in relation to hormone receptor status.

**Methods:**

Paraffin-embedded tumor material was collected from 480 early-stage breast cancer patients younger than 41 years who participated in one of four European Organization for Research and Treatment of Cancer trials. Using immunohistochemistry on the whole series of tumors, we assessed estrogen receptor (ER) status and progesterone receptor (PgR) status in a standardized way. Endpoints in this study were overall survival (OS) and distant metastasis-free survival (DMFS). The median follow-up period was 7.3 years.

**Results:**

Overall, patients with ER-positive tumors had better OS rates (hazard ratio [HR] 0.63; *P *= 0.02) compared with those with ER-negative tumors. However, in the subgroup of patients who received chemotherapy, no significant difference in OS (HR 0.87; *P *= 0.63) and DMFS (HR 1.36; *P *= 0.23) was found between patients with ER-positive tumors or those with ER-negative tumors. These differences were similar for PgR status.

**Conclusion:**

Young patients with hormone receptor-positive tumors benefit less from adjuvant systemic chemotherapy than patients with hormone receptor-negative tumors. These results confirm that chemotherapy alone cannot be considered optimal adjuvant systemic treatment in breast cancer patients 40 years old or younger with hormone receptor-positive tumors.

## Introduction

Breast cancer in premenopausal women is associated with worse outcome compared with postmenopausal patients [1]. Approximately 7% of women diagnosed with breast cancer are younger than 40 years old [2]. Very young women (that is, younger than 35 years old), especially, are at a high risk of developing distant metastases. Therefore, they are recommended to receive adjuvant systemic chemotherapy regardless of tumor stage or grade [3]. In addition, high local regional recurrence rates after breast-conserving therapy have been reported in young premenopausal patients with breast cancer [4]. Although it is clear that young age is an independent prognosticator of adverse outcome in breast cancer, controversies regarding the optimal treatment in this population exist.

Adjuvant systemic chemotherapy in premenopausal patients has been shown to improve survival [1], but controversy about the role of chemotherapy in patients with hormone receptor-positive tumors still exists. Aebi and colleagues [5] clearly showed that the endocrine effects of chemotherapy alone might not be sufficient for very young patients with breast cancer. In this study, it was shown that estrogen receptor (ER)-positive tumors in patients younger than 35 years and treated with cyclophosphamide, methotrexate, and 5-fluorouracil (CMF) had a significantly worse disease-free survival compared with ER-negative patients.

To detect whether we could confirm these data by finding similar results, we studied the efficacy of chemotherapy in young patients with breast cancer according to ER status and progesterone receptor (PgR) status and selected patients 40 years old or younger at the time of primary diagnosis from four European Organization for Research and Treatment of Cancer (EORTC) trials that were conducted by the EORTC Breast Cancer Group and Radiotherapy Group.

## Materials and methods

Data were collected from four EORTC trials. In total, 9,938 patients participated in these trials; 934 of these patients were 40 years old or younger at the time of diagnosis. The trial designs are summarized below:

EORTC trial 10801 (1980 to 1986, median follow-up 13.4 years) was conducted to assess the safety of breast-conserving treatment. In this trial, patients were randomly assigned between breast-conserving surgery combined with radiotherapy and radical mastectomy. Six cycles of adjuvant chemotherapy with cyclophosphamide 100 mg/m^2 ^given orally on days 1 to 14, methotrexate 40 mg/m^2 ^given intravenously on days 1 and 8, and 5-fluorouracil 600 mg/m^2 ^(CMF) given intravenously on days 1 and 8 were indicated for all patients under the age of 55 with positive nodes. A total of 902 patients were randomly assigned [6].

EORTC trial 10854 (1986 to 1991, median follow-up 10.8 years) studied the question of whether one course of perioperative chemotherapy given directly after surgery yields better results in terms of treatment outcome than surgery alone. Perioperative chemotherapy consisted of a single course of doxorubicin 50 mg/m^2^, 5-fluorouracil 600 mg/m^2^, and cyclophosphamide 600 mg/m^2 ^(FAC) administered intravenously within 36 hours after surgery. For axillary lymph node-positive premenopausal patients in the perioperative chemotherapy group, adjuvant chemotherapy consisting of five cycles of CMF was recommended. For node-positive patients younger than 50 years who did not receive perioperative chemotherapy, one conventional course of FAC followed by five cycles of CMF after surgery was recommended. Postmenopausal patients were recommended to receive tamoxifen. A total of 2,795 patients were included [7].

EORTC trial 10902 (1991 to 1999, median follow-up 6.1 years) was set up to determine the value of preoperative chemotherapy. Patients were randomly assigned to receive four cycles of chemotherapy either before or after surgery. Chemotherapy consisted of four cycles of 5-fluorouracil 600 mg/m^2^, epirubicin 60 mg/m^2^, and cyclophosphamide 600 mg/m^2 ^(FEC) administered intravenously at 3-weekly intervals. In the preoperative chemotherapy group, surgical therapy followed within 4 weeks of the fourth course of chemotherapy. In the postoperative chemotherapy group, the first cycle was given within 36 hours after surgery. Patients not younger than 50 years received tamoxifen for 2 years. A total of 698 patients were randomly assigned [8].

EORTC trial 22881 (1989 to 1996, median follow-up 5.1 years) was conducted to study the effect of an additional dose of 16 Gy radiation to the tumor bed among early-stage breast cancer patients who received 50 Gy radiotherapy after lumpectomy. Patients with a microscopically incomplete resection were assigned to receive boost doses of 10 or 26 Gy. Premenopausal patients with axillary lymph node involvement received six cycles of adjuvant CMF, and all postmenopausal patients received tamoxifen 20 mg per day during at least 2 years. A total of 5,569 patients were enrolled [9].

In all trials, if adjuvant chemotherapy was indicated, patients received either CMF or an anthracyclin-based regimen (FAC or FEC). Adjuvant hormonal therapy for premenopausal hormone receptor-positive patients was not yet recommended at the time these trials were conducted. In the two oldest trials, tamoxifen administration was not even recorded. This explains the high number of patients for whom no information was found on tamoxifen use. In the trials in which tamoxifen use was recorded, less than 5% of patients 41 years old or younger received tamoxifen. Therefore, we have to assume that only a very small fraction of the patient population in this study received tamoxifen.

### Hormone receptor staining

Paraffin-embedded tumor material was collected from 480 patients 40 years old or younger. Tumors were histologically graded using hematoxylin and eosin slides as described previously [10]. Immunohistochemical staining for ER and PgR status was performed using a tissue microarray [11-14]. Three core biopsies were taken from each tumor block and inserted into a donor block. Immunohistochemical staining for ER was performed using the monoclonal antibody DAKO-ER, 1D5 (DakoCytomation Denmark A/S, Glostrup, Denmark), for PgR using the monoclonal antibody mPRI (TRANSBIO, Paris, France). Immunohistochemical staining was scored using a semiquantative system based on the percentage of positive nuclei. After the percentage of positive nuclei in three core biopsies was counted, the mean value was taken. For both ER and PgR, tumors with greater than 10% of the tumor cells showing nuclear staining were considered positive.

### Statistical analysis

Analyses were performed for distant metastasis-free survival (DMFS) and overall survival (OS). DMFS was defined as the interval from time of randomization to time of distant metastasis or death, whichever came first. OS was defined as time from randomization to death from any cause. Survival curves were estimated using the Kaplan-Meier method [15]. Differences in survival were analyzed using Cox proportional hazard models [16]. The statistical analyses were performed using SPSS software (SPSS Inc., Chicago, IL, USA). A direct comparison of patients who received chemotherapy versus those who did not receive chemotherapy was not feasible. (This would have introduced a selection bias in this retrospective analysis as the vast majority of patients receiving chemotherapy had positive axillary lymph nodes.) Therefore, conclusions in this explorative analysis were based on indirect comparisons.

## Results

### Participants

Paraffin-embedded tumor specimens were collected from 480 patients 40 years old or younger at the time of diagnosis (Table 1). For 12 patients, ER status could not be scored, and for 16 patients, PgR status could not be scored. Positive ER status and positive PgR status were found in 288 and 223 patients, respectively. Two hundred patients received prolonged adjuvant systemic chemotherapy, whereas 279 patients did not receive adjuvant systemic chemotherapy. Of the patients not receiving chemotherapy, 94% were node-negative; 85% of patients who did receive chemotherapy were node-positive. Characteristics related to adjuvant systemic chemotherapy treatment are listed in Table 2. At the time of the analysis, the median follow-up was 7.3 years, 106 (22%) patients had died, and 155 (32%) patients developed a distant recurrence or died. The distribution of events stratified by ER status is listed in Table 3.

**Table 1:** Characteristics of 480 breast cancer patients 40 years old or younger

Characteristic		Number of patients (percentage) Total = 480
Clinical tumor size	T1	185 (39)
	T2/3	293 (61)
	Missing data	2 (0)
Histological tumor size	T1	292 (61)
	T2/3	151 (31)
	Missing data	37 (8)
Histological nodal status	Negative	288 (60)
	Positive	188 (39)
	Missing data	4 (1)
Surgery	Breast-conserving surgery	393 (82)
	Mastectomy	86 (18)
	Missing data	1 (0)
Adjuvant chemotherapy	No	279 (58)
	Yes	200 (42)
	Missing data	1 (0)
Tamoxifen^a^	No	273 (57)
	Yes	9 (2)^b^
	Missing data	198 (41)
Histological grade	I	70 (15)
	II	145 (30)
	III	255 (53)
	Missing data	10 (2)
Estrogen receptor	Positive	288 (60)
	Negative	180 (38)
	Missing data	12 (3)
Progesterone receptor	Positive	223 (46)
	Negative	241 (50)
	Missing data	16 (3)

^a^During the period of time in which these trials were conducted, tamoxifen was not routinely given to premenopausal patients with estrogen receptor (ER)-positive tumors. ^b^All patients who received tamoxifen had ER-positive tumors. T, tumor size.

**Table 2:** Patient and tumor characteristics specified by adjuvant chemotherapy^a^

Characteristic	Number of patients (percentage)	
	No adjuvant chemotherapy Number = 279	Adjuvant chemotherapy Number = 200

ER-positive	161 (58)	126 (63)
Anthracycline-based		66
CMF		60
ER-negative	110 (39)	70 (35)
Anthracycline-based		48
CMF		22
PgR-positive	135 (48)	88 (44)
PgR-negative	135 (48)	105 (53)
T1	187 (67)	105 (53)
T2/T3	76 (27)	75 (38)
Node-negative	259 (93)	29 (15)
Node-positive	18 (6)	170 (85)
Breast-conserving surgery	247 (89)	146 (73)
Mastectomy	32 (11)	53 (27)

^a^Missing data not shown. CMF, cyclophosphamide, methotrexate, and 5-fluorouracil; ER, estrogen receptor; PgR, progesterone receptor; T, tumor size.

**Table 3:** Distribution of events according to ER status and chemotherapy^a^

	Number of events (percentage)	
	No adjuvant chemotherapy	Adjuvant chemotherapy

Deaths (number of events = 106)		
ER-positive	19 (18)	35 (33)
ER-negative	29 (27)	19 (18)
Distant metastasis or death (number of events = 155)		
ER-positive	37 (24)	54 (35)
ER-negative	38 (25)	21 (14)

^a^Missing data not shown. ER, estrogen receptor.

### Overall results

#### Estrogen receptor status

**Figure 1:**
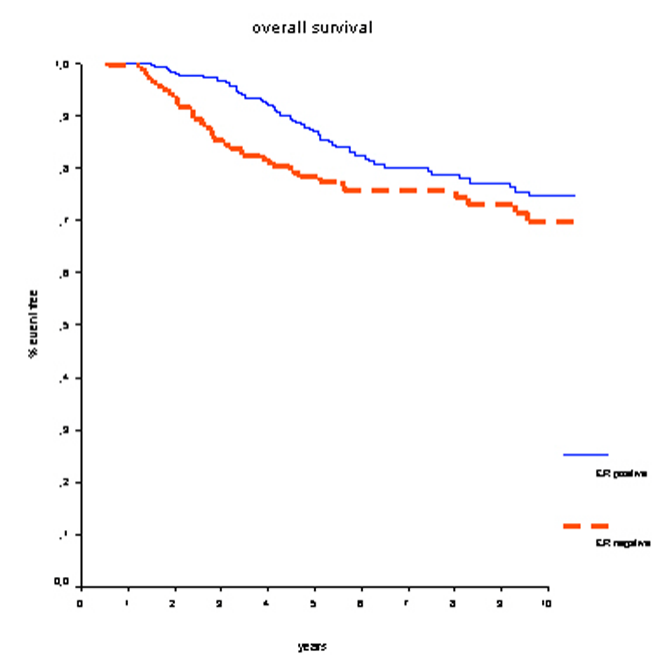
Overall survival for all patients. Estrogen receptor (ER)-positive patients have a better prognosis.

**Figure 2:**
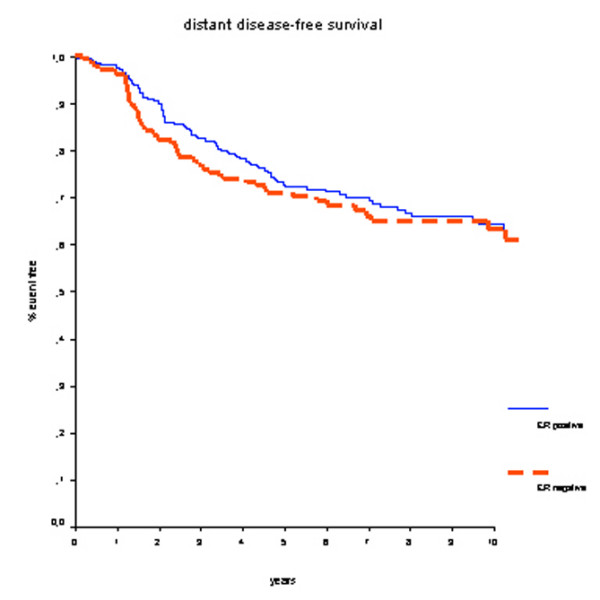
Distant metastasis-free survival for all patients. There is no difference in prognosis according to estrogen receptor (ER) status.

Overall, patients with ER-positive tumors had better OS rates compared with ER-negative patients (hazard ratio [HR] 0.63, 95% confidence interval (CI) 0.43 to 0.93; *P *= 0.02) (Figure 1). Survival rates at 7 years were 82% for the ER-positive group and 77% for the ER-negative group. DMFS rates were 70% for the ER-positive group and 66% for the ER-negative group (HR 0.90, 95% CI 0.65 to 1.24; *P *= 0.51) (Figure 2).

#### Progesterone receptor status

PgR status yielded similar results: patients with progesterone-positive tumors had better OS (HR 0.59, 95% CI 0.40 to 0.88; *P *= 0.01). However, for DMFS this difference was not of statistical significance (HR 0.78, 95% CI 0.57 to 1.01; *P *= 0.14).

### Patients who did not receive prolonged adjuvant chemotherapy

#### Estrogen receptor status

**Figure 3:**
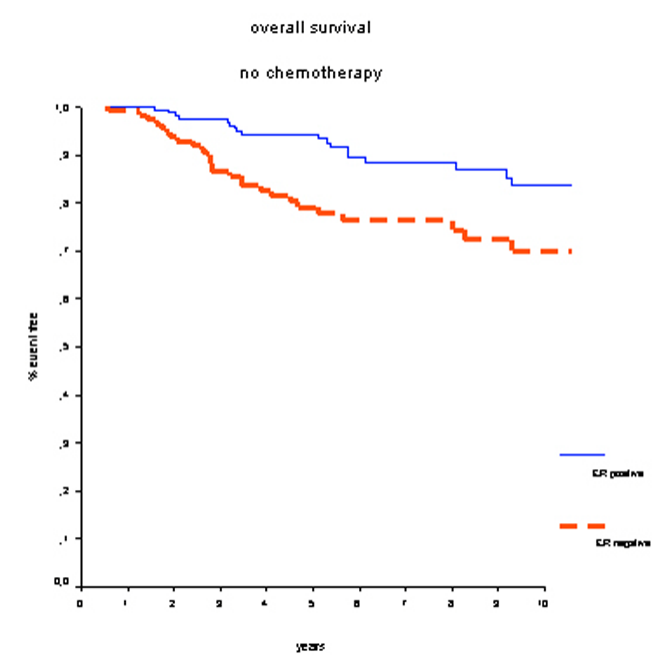
Overall survival in patients who did not receive adjuvant chemotherapy. Estrogen receptor (ER)-positive patients have a better prognosis.

**Figure 4:**
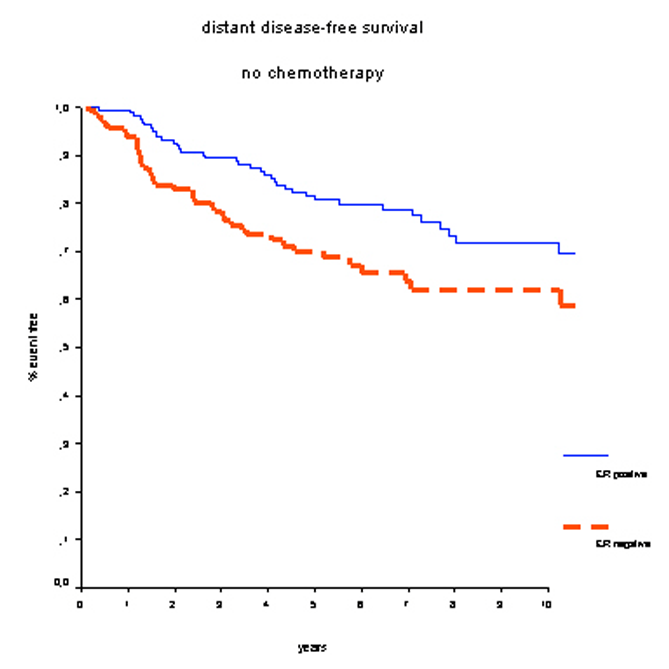
Distant metastasis-free survival in patients who did not receive adjuvant chemotherapy. Estrogen receptor (ER)-positive patients have a better prognosis.

In patients who did not receive adjuvant systemic chemotherapy, positive ER status was associated with better OS (HR 0.41, 95% CI 0.23 to 0.74; *P *< 0.01) (Figure 3). Survival rates at 7 years were 90% for the ER-positive group and 77% for the ER-negative group. Also, DMFS rates at 7 years were significantly better for ER-positive patients: 80% versus 64% (HR 0.59, 95% CI 0.37 to 0.92; *P *= 0.02) (Figure 4).

#### Progesterone receptor status

In patients who did not receive adjuvant systemic chemotherapy, positive PgR status was associated with better OS (HR 0.44, 95% CI 0.24 to 0.80; *P *< 0.01). Survival rates at 7 years were 88% for the PgR-positive group and 75% for PgR-negative group. DMFS rates at 7 years were 79% for PgR-positive patients and 67% for PgR-negative patients (HR 0.66, 95% CI 0.42 to 1.04; *P *= 0.07).

### Patients who received prolonged adjuvant systemic chemotherapy

#### Estrogen receptor status

**Figure 5:**
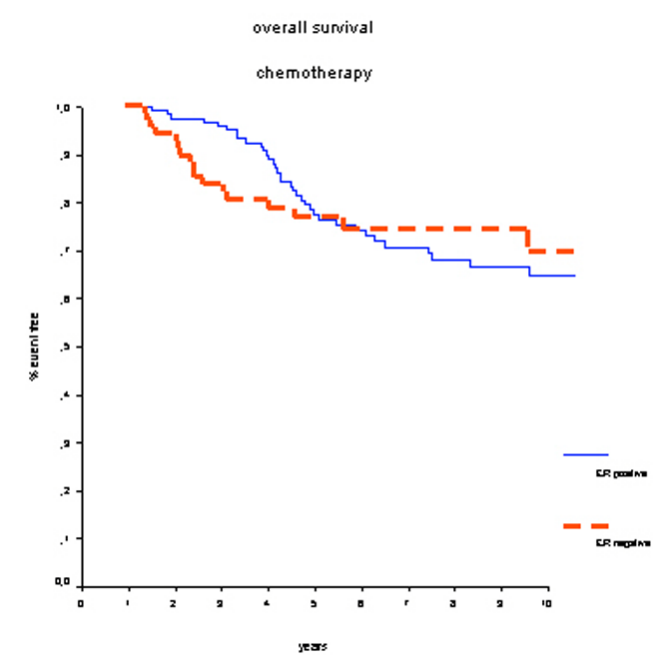
Overall survival in patients who received adjuvant chemotherapy. There is no long-term survival benefit for estrogen receptor (ER)-positive patients. The crossing lines could be explained by the initial beneficial effect of chemotherapy-induced amenorrhea.

**Figure 6:**
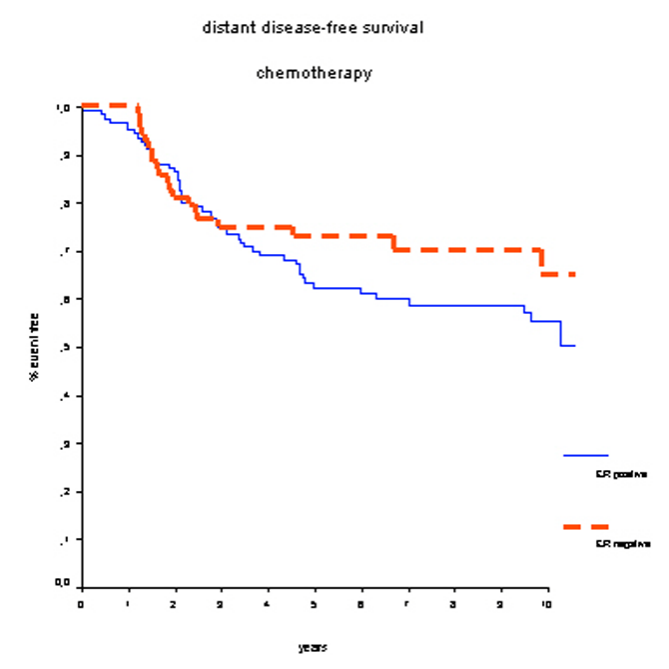
Distant metastasis-free survival in patients who received adjuvant chemotherapy. There is no statistically significant difference in prognosis according to estrogen receptor (ER) status.

In the group of 200 patients who did receive adjuvant systemic chemotherapy, treatment outcome was not significantly different between ER-positive and ER-negative breast cancer patients. Survival rates at 7 years were 70% for the ER-positive group and 75% for the ER-negative group (HR 0.87, 95% CI 0.50 to 1.52; *P *= 0.63) (Figure 5), and DMFS rates were 59% for the ER-positive group and 70% for the ER-negative group (HR 1.36, 95% CI 0.82 to 2.26; *P *= 0.23) (Figure 6). No further subgroup analyses specified by type of chemotherapy were performed since these groups would have had insufficient numbers and events.

#### Progesterone receptor status

According to PgR status, no difference in treatment outcome for patients who have received adjuvant systemic chemotherapy was found. In both the PgR-positive and PgR-negative patient groups, the survival rate at 7 years was 72% (HR 0.84, 95% CI 0.49 to 1.43; *P *= 0.51). Also, DMFS rates did not differ significantly between the PgR-positive group (59%) and the PgR-negative group (64%) (HR 1.02, 95% CI 0.65 to 1.60; *P *= 0.93).

### Multivariate analysis

Multivariate Cox regression OS analyses were performed separately for ER status and PgR status. Other covariates included nodal status, tumor size, and the administration of prolonged adjuvant chemotherapy. Both ER status (relative risk [RR] 1.65) and PgR status (RR 1.56; data not shown) remained independent prognostic factors with a significant impact on OS (Table 4).

**Table 4:** Multivariate Cox regression analysis of overall survival

Characteristic	Relative risk	95% confidence interval	*P *value
ER-negative	1.65	1.09–2.50	0.02
Node-positive	1.70	0.79–3.66	0.17
Tumor size >2 cm	1.66	1.09–2.52	0.02
Adjuvant chemotherapy	1.02	0.48–2.17	0.96

ER status remains an independent prognostic factor of overall survival. ER, estrogen receptor.

## Discussion

This pooled analysis of patients 40 years old or younger demonstrated that hormone receptor-positive patients experienced no survival advantage of prolonged adjuvant CMF chemotherapy compared with hormone receptor-negative patients. However, in patients who did not receive adjuvant chemotherapy, hormone receptor-positive status was associated with improved survival rates compared with hormone receptor-negative status. In overall multivariate analyses, both ER-positive status and PgR-positive status remained independent prognostic factors of OS.

Our study has some limitations. First, the current analysis retrospectively uses heterogeneous data from different randomized trials. Second, adjuvant CMF chemotherapy to a large extent has been replaced by anthracycline-containing chemotherapeutic regimens because of higher treatment efficacy in patients with breast cancer regardless of hormone receptor or menopausal status [1]. Also, taxanes are increasingly being used, showing additional survival benefits. Therefore, different effects might have been demonstrated when newer chemotherapy regimens were used throughout the included studies. Third, the direct comparison between administration of chemotherapy versus no chemotherapy in hormone receptor-positive and hormone receptor-negative patients would have been very interesting. However, the confounding effect of axillary lymph node status would have introduced a significant selection bias because the majority of patients who received chemotherapy had positive axillary lymph nodes. Nevertheless, in multivariate analysis including axillary lymph node status, tumor size, and the administration of prolonged adjuvant chemotherapy, hormone receptor status remained an independent prognostic factor for OS. Fourth, the survival curves of the ER-positive and ER-negative group depicted in Figure 5 (overall survival in patients receiving chemotherapy) are crossing. This implies that that the proportional hazards assumption is not justified. The rapid decrease in survival benefit after a couple of years in the ER-positive group may well be explained by the chemotherapy-induced amenorrhea and the associated low estrogen levels. Unfortunately, no information on the number of patients who have become amenorrheatic could be retrieved in order to test this hypothesis. Despite these limitations, this pooled analysis of four randomized controlled trials used individual patient data with renewed pathological analysis of hormone receptor status. Because less than 5% of the study population received tamoxifen, the effect of chemotherapy alone in hormone receptor-positive patients could be well studied. By analyzing hormone receptor status centrally, we have provided standardized measurements for all tumors in the study.

Adjuvant systemic chemotherapy is a well-established treatment modality in premenopausal breast cancer. In patients 35 years old or younger, administration of chemotherapy is advocated regardless of nodal status, tumor size, or grade [3]. However, the efficacy of chemotherapy in premenopausal patients with ER-positive breast cancer has been questioned [5,17,18]. Our findings are in accordance with data from Aebi and colleagues [5], who demonstrated that young premenopausal patients with breast cancer treated with adjuvant CMF chemotherapy had a higher risk of relapse and death than older premenopausal patients, especially if their tumors were ER-positive. In addition, several neoadjuvant chemotherapy studies have demonstrated that patients with ER-negative tumors are more likely to achieve a pathological complete response than those with ER-positive tumors [19-21]. Moreover, these studies found that when patients with ER-negative tumors achieved a pathological complete response their survival was comparable with that of ER-positive patients.

To optimize adjuvant systemic treatment in premenopausal patients with breast cancer, several investigators have studied the role of ovarian suppression by luteinizing hormone-releasing hormone (LHRH) agonists. Recently, the Early Breast Cancer Overview group reported a meta-analysis of individual patient data on the use of LHRH agonists [22]. When chemotherapy alone was compared with chemotherapy in combination with an LHRH agonist, a difference between younger and older premenopausal women with hormone receptor-positive disease was found. In patients 40 years old or younger, the addition of an LHRH agonist significantly reduced the risk of recurrence and death (HR 0.74; *p *= 0.01). This effect was greatest in the group 35 years old or younger, whereas in the group older than 40 years, the addition of an LHRH agonist did not improve outcome. When chemotherapy alone was compared with LHRH agonist with or without tamoxifen in younger premenopausal patients with hormone receptor-positive tumors, the endocrine therapy improved outcome (mortality HR 0.82; *P *= 0.15). Conversely, in hormone receptor-negative patients, the same comparison significantly favored treatment with chemotherapy (62.1% increased rate of recurrence or death; *P *= 0.003). To date, no trial has compared an LHRH agonist against chemotherapy with tamoxifen in both arms. This relevant and important issue needs to be resolved. Although these results underline the fact that chemotherapy may be equivalent to hormonal ovarian suppression in terms of treatment outcome in hormone receptor-positive patients, these results firmly demonstrate a beneficial effect of LHRH agonists as additional therapy, especially in young patients with breast cancer.

Three important ongoing trials are specifically investigating ovarian function suppression (Suppression of Ovarian Function Trial, or SOFT), an aromatase inhibitor (Tamoxifen and EXemestane Trial, or TEXT), and the need for chemotherapy (Premenopausal Endocrine Responsive CHEmotherapy, or PERCHE) in adjuvant treatment for young patients with hormone receptor-positive breast cancer [23].

The 2005 St. Gallen Consensus Committee on adjuvant therapy for early-stage breast cancer recommended that the first consideration in treatment selection be endocrine responsiveness [24]. Three categories are identified: endocrine-responsive, endocrine-nonresponsive, and tumors of uncertain endocrine responsiveness. These categories refer to the groups of tumors that are responsive to endocrine therapies alone, chemotherapy alone, and chemotherapy and endocrine therapy combinations, respectively. The 2005 Panel viewed tamoxifen as a standard adjuvant treatment for premenopausal endocrine-responsive patients. The combination of tamoxifen with an LHRH agonist is recommended for very young patients, especially in intermediate- and high-risk groups, and for premenopausal patients of any age at high risk, especially if chemotherapy did not induce amenorrhea. The use of aromatase inhibitors in premenopausal patients is not recommended outside of clinical trials, except when tamoxifen is contraindicated, especially in node-positive disease. Chemotherapy in addition to hormone therapy is advised for endocrine-responsive patients with node-positive disease.

## Conclusion

In this retrospective pooled analysis of four studies using heterogeneous chemotherapy regimens, we have demonstrated that treatment efficacy of adjuvant chemotherapy is less in young patients with hormone receptor-positive tumors compared with young patients with hormone receptor-negative tumors. Therefore, we conclude that chemotherapy alone is not a sufficient systemic treatment strategy in young patients with hormone receptor-positive breast cancer. Hormone responsiveness is the key for tailoring therapy for young patients with breast cancer.

## Abbreviations

CI = confidence interval; CMF = cyclophosphamide, methotrexate, and 5-fluorouracil; DMFS = distant metastasis-free survival; EORTC = European Organization for Research and Treatment of Cancer; ER = estrogen receptor; FAC = doxorubicin, 5-fluorouracil, and cyclophosphamide; FEC = 5-fluorouracil, epirubicin, and cyclophosphamide; HR = hazard ratio; LHRH = luteinizing hormone-releasing hormone; OS = overall survival; PgR = progesterone receptor; RR = relative risk.

## Competing interests

JAvdH was a research fellow at the EORTC from 1999 to 2000. The other authors declare that they have no competing interests.

## Authors' contributions

JAvdH was responsible for the acquisition of data and analysis and interpretation of the data and drafted the manuscript. JSDM and MJvdV were involved in drafting the manuscript and revised it critically for important intellectual content. CJHvdV revised the manuscript critically for important intellectual content and gave final approval of the version to be published. All authors read and approved the final manuscript.
